# Effect of vosoritide on spine morphology in children with achondroplasia: 1-year results from a randomized phase 2 study

**DOI:** 10.1210/jendso/bvag008

**Published:** 2026-02-20

**Authors:** Melita Irving, Ravi Savarirayan, Julie E Hoover-Fong, Anne Dee, Swati Mukherjee, Christine Rivat, Ian Sabir, Klane K White

**Affiliations:** Guy's and St. Thomas' NHS Foundation Trust, Evelina Children's Hospital, London SE1 7EH, UK; Murdoch Children's Research Institute, Royal Children's Hospital, University of Melbourne, Parkville, Victoria 3052, Australia; Johns Hopkins University, Baltimore, MD 21218, USA; BioMarin Pharmaceutical Inc., Novato, CA 94949, USA; BioMarin (UK) Ltd, London WC1A 2SL, UK; BioMarin (UK) Ltd, London WC1A 2SL, UK; BioMarin (UK) Ltd, London WC1A 2SL, UK; Children's Hospital Colorado, Aurora, CO 80045, USA

**Keywords:** achondroplasia, growth, kyphosis, spinal stenosis, spine, vosoritide

## Abstract

Achondroplasia is a skeletal dysplasia condition caused by reduced endochondral ossification resulting in disproportionate short stature and skeletal deformities, including thoracolumbar kyphosis (TLK) and spinal stenosis. Vosoritide, the first and only approved targeted therapy for achondroplasia, increases bone growth, but its impact on spinal morphology has not been assessed. The randomized, double-blind, placebo-controlled phase 2 CANOPY ACH-2I study (111-206; NCT03583697) evaluated the safety and efficacy of vosoritide in 75 children aged 0 to <5 years. Interpedicular distance (IPD), sagittal width of the lumbar spinal canal, and TLK angle were measured on spinal radiographs taken at baseline and 1 year after vosoritide or placebo treatment. Differences in least-squares mean (LSM) change from baseline (95% CI) between treatment groups were determined with an analysis of covariance model. Measurable improvements in IPD and spinal canal width across L1 to L5 were observed with vosoritide compared with placebo after 1 year. L4 was the most impacted by vosoritide for IPD (LSM difference [95% CI], 0.509 [−0.034 to 1.052] mm, *P* = .066) and canal width (1.433 [0.547 to 2.320] mm, *P* = .002). Vosoritide treatment also reduced the natural increase in TLK angle in children 0 to <0.5 years and provided greater improvements in children ≥0.5 to <5 years. After 1 year, fewer children treated with vosoritide (33.3%) vs placebo (59.3%) had pathological (≥20°) TLK angles (*P* = .037). These preliminary results suggest that early vosoritide treatment may improve spinal morphology and reduce the risk of spinal stenosis in children with achondroplasia.

Clinical Trial Information: NCT03583697.

Achondroplasia is a rare skeletal dysplasia condition caused by overactivation of the fibroblast growth factor receptor 3 (FGFR3) signaling pathway due to a gain-of-function variant in *FGFR3*, resulting in impaired endochondral bone growth and disproportionate short stature [1, 2]. Children with achondroplasia experience reduced annualized growth velocity (AGV; cm/year) apparent by 3 months of age on average, a final height 6 to 7 SDs below average-stature peers, and disproportionality marked by increased upper-to-lower body segment ratios [1, 3]. Additional complications of reduced bone growth include spinal deformities, foramen magnum stenosis, central and obstructive sleep apnea, recurrent ear infections, and genu varum [1, 2]. Thoracolumbar kyphosis (TLK) and symptomatic spinal stenosis are 2 common spinal complications of achondroplasia that cause long-term pain, impaired mobility, and require life-long management [2, 4]. These complications often require surgical intervention in children and adults [2].

Normal development of the spine and thoracic cage requires a synchronized and rapid succession of growth events to maintain proper body segment proportions [5]. The neurocentral synchondrosis, a growth plate at the junction of the pedicle and vertebral body, is particularly important for growth of the vertebral body and posterior arch and is typically fused by 9 years of age [5]. In individuals with achondroplasia, disrupted bone ossification causes truncated and thickened spinal pedicles, leading to narrowing of the spinal canal [6-8].

Pathological TLK (≥20° sagittal deformity measured on a lateral radiograph) is common in infants with achondroplasia and is associated with delayed motor development [9, 10]. Furthermore, TLK is exacerbated at the sitting stage of development due to truncal hypotonia and a disproportionate, large head [7, 10, 11]. Pathological TLK begins to resolve with the onset of ambulation in most children with achondroplasia, with a decrease in prevalence to 33% by 3 years of age [9, 11]. Conversely, TLK persists in ∼23% of adolescents (10 to 20 years of age) with achondroplasia [9]. Children with persistent TLK are at increased risk for developing symptomatic spinal stenosis that will require surgical intervention later in life [11, 12].

Spinal stenosis is commonly defined by progressive narrowing of the interpedicular distance (IPD) seen on anterior/posterior (A/P) radiographs and reduced sagittal dimensions of the vertebral bodies seen on lateral radiographs [13]. While spinal stenosis can occur at all vertebral levels, symptomatic lumbar stenosis is most prevalent in individuals with achondroplasia [8]. Spinal stenosis can become symptomatic in the second decade of life, with the narrowing of the spinal canal causing compression of the normal-sized spinal cord and nerve roots [12, 14]. Spinal stenosis in individuals with achondroplasia can cause neurogenic claudication and radiculopathy, with common symptoms including walking intolerance, bowel or bladder dysfunction, pain in the lower limbs, cauda equina syndrome, and paraplegia [11, 12, 15].

Surgical intervention during childhood is usually successful in relieving the symptoms of spinal stenosis but may result in postoperative complications such as a dural tear, postoperative junctional kyphosis, instrumentation failure, and deep surgical-site infection [16, 17]. Moreover, it has been reported that up to 18% of patients require additional operations [16]. The spinal canal reaches 95% of its final size by 5 years of age in average-stature children and even earlier in children with achondroplasia [5, 18]. Therefore, the opportunity for prevention and correction of spinal deformities is reduced in children with achondroplasia, and early intervention is critical.

Vosoritide, a C-type natriuretic peptide analog, is a potent stimulator of endochondral bone growth and is the first approved targeted treatment for achondroplasia indicated in infants and children until the closure of the epiphyses [19, 20]. In an expansive clinical trial program, vosoritide persistently improved height *Z* scores and AGV compared with untreated children with achondroplasia [21, 22]. Vosoritide also improved body proportionality and health-related quality of life [21, 23]. These clinical results are reflected in real-world evidence, which has demonstrated the positive effects of vosoritide on overall skeletal growth and functionality in children with achondroplasia [24-26]. However, clinical evidence demonstrating the effect of vosoritide on spinal morphology is limited. Here, we examine key parameters of spinal morphology in children with achondroplasia treated with vosoritide or placebo for 1 year beginning <5 years of age in the phase 2 CANOPY ACH-2I clinical study (111-206).

## Materials and methods

### Study design

The detailed study design of the double-blind, randomized, placebo-controlled phase 2 CANOPY ACH-2I trial (111-206; NCT03583697) has been published previously [22]. Briefly, children 0 to <5 years of age with genetically confirmed achondroplasia were enrolled into 3 sequential cohorts consisting of participants aged 2 to <5, 0.5 to <2, and <0.5 years at the time of screening. Exclusion criteria included concurrent endocrine, short stature, or cardiac conditions; evidence or history of cervicomedullary spinal cord compression; and previous or planned limb-lengthening procedures. Participants were randomly assigned to receive daily subcutaneous injections of vosoritide (30 µg/kg for participants aged <2 years and 15 µg/kg for participants aged ≥2 years) or placebo for 52 weeks. The randomization schedule, which did not include radiographic measures as a factor, was developed by an independent vendor, and the study sponsor and study-site personnel were blinded to treatment assignments.

### Outcome assessments

**Figure 1:**
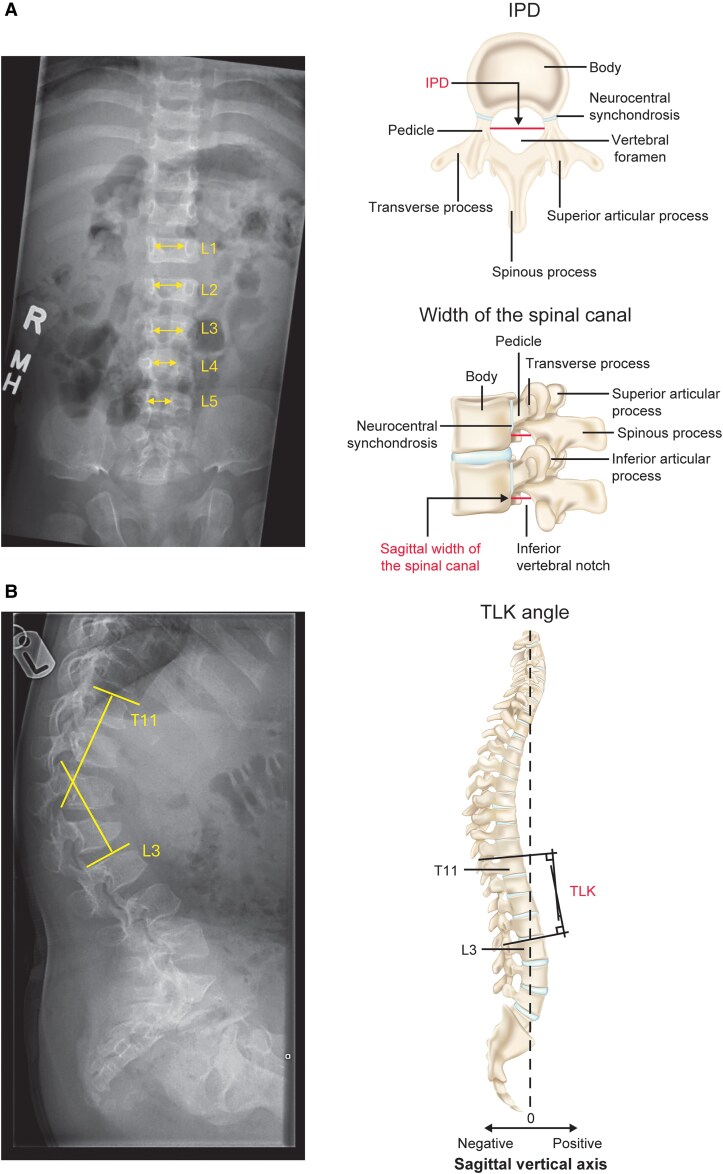
Representative (A) A/P radiographic of the lumbar spine in a child with achondroplasia and schematic of IPD and spinal canal width assessments and (B) lateral radiographic displaying TLK in a child with achondroplasia and schematic of the TLK angle assessment. Arrows in the A/P representative radiograph represent IPD of L1 to L5. Measurement angle annotations in the lateral radiograph are for representative illustrations only. Abbreviations: A/P, anterior/posterior; IPD, interpedicular distance; L1 to L5, lumbar vertebrae 1 to 5; T, thoracic vertebra; TLK, thoracolumbar kyphosis.

Spinal morphology was assessed using lateral and A/P radiographs from all participants in CANOPY ACH-2I who had both baseline and week 52 measurements available per morphological parameter (Fig. 1). Image acquisition was standardized across the study sites, and images were centrally read by independent radiologists to minimize variability in measurements. As depicted in Fig. 1, IPD was defined as the distance between medial aspects of both pedicles. Sagittal width of the spinal canal was measured at the inferior level of the pedicle. Thoracolumbar angles were measured from the top of thoracic vertebra 11 (T11) to the top of lumbar vertebra 3 (L3).

### Ethics

This study was conducted according to the Declaration of Helsinki, and the independent ethics committee or institutional review boards of all study sites approved the study protocol. The legally authorized representatives (parents/guardians) of all participants provided written informed consent.

### Statistics

To adjust for differences in demographics and baseline characteristics, least-squares mean (LSM) changes from baseline were calculated using an analysis of covariance model that included treatment arm, sex, randomization age strata, baseline age, baseline height *Z* score, baseline AGV, and baseline morphological measure (IPD, sagittal width, or TLK) as model terms. Least-squares mean differences and 95% CIs between treatment groups were based on the same model.

## Results

### Participants

**Figure 2:**
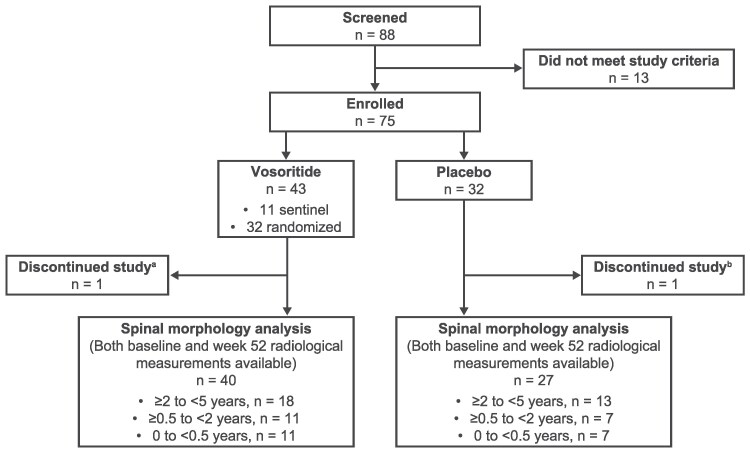
CONSORT diagram of study participants. Abbreviations: AE, adverse event. ^a^One participant who received vosoritide treatment discontinued due to a fatal AE assessed as not related to study treatment. ^b^One participant who received placebo discontinued from study due to withdrawal by participant.

Of the 75 participants enrolled in CANOPY ACH-2I, 43 received vosoritide (including 11 nonrandomized sentinels) and 32 received placebo (Fig. 2). Spinal morphology assessments at both baseline and week 52 were available for 67 participants. Most participants were White and not Hispanic or Latino (Table 1). Across all ages, 44.4% vs 57.5% of participants treated with placebo vs vosoritide were male. Baseline AGV was greater in the younger age groups, as expected. Baseline AGV and height *Z* scores were comparable between participants in the placebo and vosoritide treatment groups.

**Table 1:** Participant demographics and baseline characteristics

	All		0 to <0.5 years		≥0.5 to <2 years		≥2 to <5 years	
	Placebo (*n* = 27)	Vosoritide (*n* = 40)	Placebo (*n* = 7)	Vosoritide (*n* = 11)	Placebo (*n* = 7)	Vosoritide (*n* = 11)	Placebo (*n* = 13)	Vosoritide (*n* = 18)
Age at day 1, years								
Mean (SD)	2.15 (1.54)	2.05 (1.48)	0.47 (0.05)	0.45 (0.05)	1.30 (0.54)	1.39 (0.45)	3.52 (0.92)	3.44 (0.95)
Median	1.93	1.88	0.49	0.47	1.34	1.42	3.31	3.20
Q1, Q3	0.50, 3.31	0.49, 3.05	0.47, 0.49	0.40, 0.49	0.81, 1.77	0.94, 1.82	2.64, 4.50	2.52, 4.26
Min, max	0.37, 4.96	0.38, 4.98	0.37, 0.50	0.38, 0.49	0.54, 1.93	0.73, 1.95	2.43, 4.96	2.12, 4.98
Sex, *n* (%)								
Male	12 (44.4)	23 (57.5)	1 (14.3)	5 (45.5)	4 (57.1)	8 (72.7)	7 (53.8)	10 (55.6)
Female	15 (55.6)	17 (42.5)	6 (85.7)	6 (54.5)	3 (42.9)	3 (27.3)	6 (46.2)	8 (44.4)
Race, *n* (%)								
White	21 (77.8)	27 (67.5)	5 (71.4)	7 (63.6)	6 (85.7)	9 (81.8)	10 (76.9)	11 (61.1)
Asian	6 (22.2)	11 (27.5)	2 (28.6)	3 (27.3)	1 (14.3)	2 (18.2)	3 (23.1)	6 (33.3)
Other	2 (7.4)	7 (17.5)	2 (28.6)	2 (18.2)	0	1 (9.1)	0	4 (22.2)
Japanese	4 (14.8)	4 (10.0)	0	1 (9.1)	1 (14.3)	1 (9.1)	3 (23.1)	2 (11.1)
Multiple	0	2 (5.0)	0	1 (9.1)	0	0	0	1 (5.6)
Ethnicity, *n* (%)								
Not Hispanic or Latino	24 (88.9)	37 (92.5)	5 (71.4)	9 (81.8)	7 (100.0)	11 (100.0)	12 (92.3)	17 (94.4)
Hispanic or Latino	3 (11.1)	3 (7.5)	2 (28.6)	2 (18.2)	0	0	1 (7.7)	1 (5.6)
AGV, cm/year								
*n*	27	40	7	11	7	11	13	18
Mean (SD)	9.84 (8.18)	11.69 (7.72)	19.89 (8.05)	22.03 (3.87)	10.55 (5.24)	11.79 (4.81)	4.05 (1.79)	5.31 (1.41)
Median	5.16	8.00	23.21	22.32	10.99	12.81	4.27	5.37
Q1, Q3	4.00, 16.06	5.37, 18.30	16.06, 24.26	19.46, 24.63	4.86, 14.72	6.90, 14.38	3.02, 5.14	4.43, 5.84
Min, max	0.3, 29.7	2.7, 30.2	4.8, 29.7	16.4, 30.2	2.5, 16.4	3.9, 18.5	0.3, 7.8	2.7, 8.7
Height *Z* score*^a^*								
*n*	27	40	7	11	7	11	13	18
Mean (SD)	−4.29 (1.52)	−3.87 (0.91)	−2.82 (0.68)	−3.42 (0.99)	−4.16 (1.49)	−3.57 (0.78)	−5.14 (1.27)	−4.33 (0.75)
Median	−4.02	−3.88	−2.87	−3.30	−4.02	−3.72	−5.12	−4.27
Q1, Q3	−5.47, −3.09	−4.39, −3.23	−3.45, −2.30	−3.90, −2.63	−5.50, −3.31	−4.01, −2.72	−5.69, −4.23	−4.70, −4.02
Min, max	−7.2, −1.5	−5.9, −2.2	−3.8, −1.9	−5.7, −2.2	−5.8, −1.5	−4.8, −2.2	−7.2, −3.0	−5.9, −3.1

Analysis includes participants with both baseline and week 52 spinal parameters. Age cohorts represent age at treatment initiation.

Abbreviations: AGV, annualized growth velocity; max, maximum; min, minimum; Q, quartile.

^*a*^Height *Z* scores were derived using age- and sex-specific reference data for average-stature children per the US Centers for Disease Control and Prevention.

### IPD and sagittal width of spinal canal

**Figure 3:**
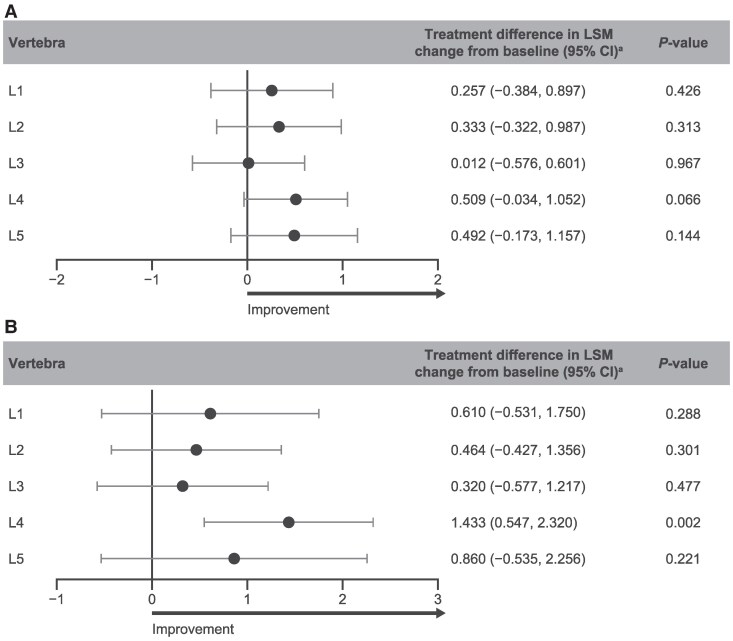
Improvement in IPD and spinal canal width with vosoritide at week 52 compared with placebo. Vosoritide vs placebo ANCOVA-adjusted treatment differences from baseline to week 52 for (A) IPD and (B) sagittal spinal canal width measurements. Differences in LSM change from baseline are reported in milimeters. Abbreviations: ANCOVA, analysis of covariance; IPD, interpedicular distance; L1 to L5, lumbar vertebrae 1 to 5; LSM, least-squares mean. ^a^Difference in LSM change was calculated as vosoritide − placebo.

Baseline mean IPD and spinal canal width were generally comparable between the vosoritide and placebo groups across all lumbar vertebrae (Table 2). Week 52 LSM change from baseline in IPD was greater in participants treated with vosoritide (range, 0.80 to 1.31 mm) than placebo (range, 0.47 to 1.06 mm) for all lumbar vertebrae. The differences in LSM change from baseline revealed numerical improvements in IPD at all lumbar vertebrae with vosoritide treatment vs placebo (Fig. 3A).

**Table 2:** LSM change from baseline in interpedicular distance and sagittal spinal canal width (mm) at L1 to L5

	Placebo				Vosoritide			
	*n*	Baseline	Week 52	LSM change from baseline (95% CI)	*n*	Baseline	Week 52	LSM change from baseline (95% CI)
Interpedicular distance (mm)								
L1	27	14.9 (2.3)	16.1 (1.6)	1.06 (0.58, 1.54)	40	15.4 (2.1)	16.6 (1.8)	1.31 (0.92, 1.70)
L2	27	14.4 (2.0)	15.3 (1.7)	0.85 (0.35, 1.34)	40	14.5 (1.8)	15.7 (1.5)	1.18 (0.78, 1.58)
L3	27	13.6 (2.0)	14.4 (1.6)	0.78 (0.34, 1.23)	40	14.0 (1.8)	14.7 (1.6)	0.80 (0.44, 1.16)
L4	27	12.6 (2.0)	13.1 (1.6)	0.47 (0.06, 0.88)	40	13.0 (1.9)	14.0 (1.8)	0.98 (0.65, 1.31)
L5	27	12.3 (2.2)	12.8 (1.6)	0.48 (−0.02, 0.98)	40	12.6 (2.0)	13.6 (2.0)	0.98 (0.57, 1.38)
Sagittal spinal canal width (mm)								
L1	26	12.5 (2.5)	12.5 (2.3)	0.06 (−0.77, 0.88)	34	12.0 (2.8)	12.7 (2.2)	0.66 (−0.05, 1.37)
L2	26	11.9 (2.4)	12.0 (1.9)	0.05 (−0.59, 0.70)	34	11.5 (2.6)	12.0 (2.3)	0.52 (−0.04, 1.08)
L3	26	11.8 (2.6)	12.2 (2.0)	0.44 (−0.21, 1.09)	34	11.3 (2.8)	12.0 (2.2)	0.76 (0.20, 1.32)
L4	26	12.3 (2.9)	12.6 (2.3)	0.19 (−0.45, 0.83)	34	11.3 (3.1)	12.9 (2.5)	1.62 (1.07, 2.18)
L5	25	13.3 (3.7)	13.9 (3.0)	0.51 (−0.49, 1.51)	31	12.4 (4.2)	13.7 (3.4)	1.37 (0.48, 2.26)

Data shown as mean (SD) unless indicated otherwise.

Abbreviations: L1 to L5, lumbar vertebrae 1 to 5; LSM, least-squares mean.

Similarly, LSM change from baseline to week 52 in spinal canal width was greater with vosoritide (range, 0.52 to 1.62 mm) compared with placebo (range, 0.05 to 0.51 mm) for all lumbar vertebrae, with differences in LSM change from baseline also revealing numerical increases in canal width in favor of vosoritide vs placebo (Table 2; Fig. 3B). Vosoritide treatment impacted L4 the most in terms of both IPD (*P* = .066) and spinal canal width increases (*P* = .002).

### TLK angle

**Figure 4:**
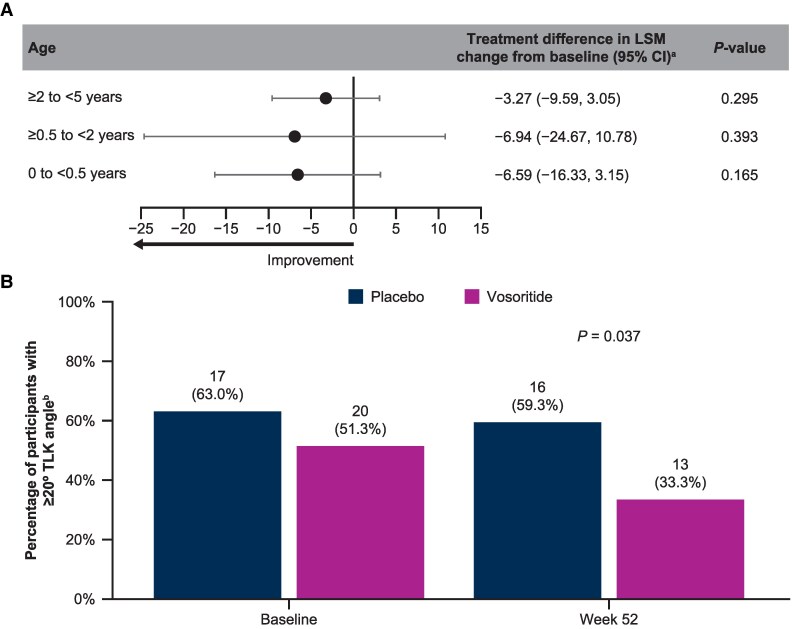
Improvement in TLK angle with vosoritide at week 52 compared with placebo. (A) Vosoritide vs placebo ANCOVA-adjusted treatment differences in TLK angle from baseline to week 52 for each age subgroup. (B) Proportion of participants with ≥20° TLK angle at week 52. Differences in LSM change from baseline are reported in degrees. *P*-value for difference in proportions of participants with pathological TLK at week 52 was calculated with Chi-square test. Abbreviations: ANCOVA, analysis of covariance; LSM, least-squares mean; TLK; thoracolumbar kyphosis. ^a^Difference in LSM change was calculated as vosoritide − placebo. ^b^Percentages were calculated using the total number of participants with both baseline and 52-week TLK angle data available (placebo, *n* = 27; vosoritide, *n* = 39 [10 sentinel]) as the denominator.

Mean TLK angle was slightly smaller in vosoritide-treated participants in the <0.5 and ≥2 to <5 year cohorts than the corresponding placebo cohorts at baseline (Table 3). As expected, TLK worsened from baseline to week 52 in the <0.5 year cohorts; however, children treated with vosoritide experienced a smaller increase in TLK angle compared with those treated with placebo (Fig. 4A). For children who enrolled aged ≥0.5 to <5 years, TLK improved in both the placebo and vosoritide groups; however, the LSM decreases in TLK angle were more pronounced with vosoritide. Consistent with these results, a smaller proportion of children treated with vosoritide (*n*/*N*, 13/39 [sentinel and randomized]; 33.3%) had a TLK angle ≥20° compared with children treated with placebo (*n/N*, 16/27; 59.3%) at week 52 across all ages of treatment initiation (*P* = .037; Fig. 4B).

**Table 3:** Difference in LSM change from baseline of TLK angle degree (top T11 to top L3) per age at treatment initiation

	Placebo				Vosoritide			
TLK angle, degree	*n*	Baseline	Week 52	LSM change from baseline (95% CI)	*n*	Baseline	Week 52	LSM change from baseline (95% CI)
Age subgroup, years								
0 to <0.5	7	27.00 (8.37)	29.71 (12.01)	9.97 (2.84, 17.11)	11	19.27 (12.69)	27.27 (10.76)	3.38 (−2.08, 8.84)
≥0.5 to <2	7	27.43 (11.41)	25.29 (16.27)	−2.27 (−13.95, 9.41)	10	27.90 (10.17)	18.60 (5.72)	−9.21 (−18.22, −0.21)
≥2 to <5	13	24.23 (17.50)	19.62 (12.80)	−2.68 (−7.26, 1.89)	18	16.50 (8.29)	11.94 (5.90)	−5.95 (−9.76, −2.15)

Data shown as mean (SD) unless indicated otherwise.

Abbreviations: CI, confidence interval; L3, lumbar vertebra 3; LSM, least-squares mean; T11, thoracic vertebra 11; TLK, thoracolumbar kyphosis.

## Discussion

These results from CANOPY ACH-2I are the first clinical trial data on the effect of vosoritide on key spinal morphological parameters in young children with achondroplasia. After 1 year, positive differences in favor of vosoritide were observed for all evaluated spinal parameters. Across all lumbar vertebrae, vosoritide treatment resulted in measurable increases in IPD and spinal canal width beyond those observed in placebo-treated children. The greatest improvements from baseline in vosoritide vs placebo-treated children were seen at L4 for both IPD and sagittal width. Together, these results suggest that vosoritide may improve growth of lumbar spine pedicles and widening of the spinal canal when treatment begins at young ages.

As expected, improvements in mean TLK angle from baseline were observed in both placebo- and vosoritide-treated children aged ≥0.5 to <2 and ≥2 to <5 years at treatment initiation. However, vosoritide treatment resulted in greater decreases in TLK angle compared with placebo. In children aged 0 to <0.5 years, when TLK angles are known to worsen with the development of hypotonia and increased head size, vosoritide appeared to reduce the progression of TLK compared with placebo [11]. Across all age cohorts, fewer children treated with vosoritide vs placebo had pathological TLK at week 52. Overall, these 1-year results suggest that increased endochondral ossification enabled by vosoritide may increase axial skeletal growth of the thoracolumbar spine in young children with achondroplasia.

Recent 1-year results from a single-center, prospective, real-world study in Japan show that vosoritide treatment slightly improved TLK and decreased exaggerated lumbar lordosis in children with achondroplasia, providing additional evidence that vosoritide treatment can positively impact axial skeletal growth and improve spinal malalignment [24]. Furthermore, children who initiated vosoritide treatment aged 0 to <0.5 years in CANOPY ACH-2I had greater increases in foramen magnum area, facial volume, and facial sinus volume at week 52 compared with those who received placebo [22]. Those results, paired with the outcomes from this analysis of the spine, suggest that vosoritide improves axial and appendicular skeletal growth when treatment begins at young ages. Early identification of individuals at risk for developing spine malformations is important to prompt early treatment and thereby maximize potential clinical benefit. Accordingly, international consensus guidelines for the implementation of vosoritide recommend initiating treatment as early as possible after diagnosis [27].

Limitations of this analysis include small cohort sizes and the relatively short follow-up period of 1 year. However, small study populations are a common limitation of trials in rare disease, and the short follow-up duration does not undermine the novelty of these findings on the impact of vosoritide on spinal morphology in children with achondroplasia. Similarly, changes in spinal morphology could not be correlated with clinical or patient-reported outcomes due to the short duration of follow-up and limitations in data collection. Longer-term analyses are needed to confirm these results. Finally, the positioning of radiographs taken from participants varied—while the variability introduced by this should be distributed randomly and evenly between treatment groups, it highlights the importance of implementing standardized methodologies for the examination of skeletal deformities across studies.

Spinal deformities often manifest with a range of neurological symptoms, including claudication, back pain, and sciatica [16, 27]. Whether the small but consistent improvements in spine growth observed after 1 year of vosoritide treatment in young children will be clinically impactful in reducing complications of symptomatic spinal stenosis—and ultimately the need for surgical correction—cannot be determined with this analysis. However, the ongoing extension study CANOPY ACH-EXT (111-208; NCT03989947) [28] will longitudinally track these study participants and further assess the impact of vosoritide on spinal morphology and the development of related complications. Continued collection of real-world data will be beneficial in determining whether documented spinal surgery rates are lower in children treated with vosoritide vs placebo and whether potential benefits of spinal growth extend into adulthood.

## Conclusion

These results suggest that initiating vosoritide treatment at a young age may positively impact spinal growth and improve spinal alignment in children with achondroplasia. Long-term follow-up of these participants will be critical in determining whether the small but consistent improvements in spine growth after 1 year persist and whether early treatment initiation reduces the rates of clinical and functional complications experienced by children with achondroplasia.

## Data Availability

The de-identified individual participant data that underlie the results reported in this article (including text, tables, figures, and appendices) will be made available together with the research protocol and data dictionaries, for noncommercial, academic purposes. Additional supporting documents may be available upon request. Investigators will be able to request access to these data and supporting documents via a data-sharing portal beginning 6 months and ending 2 years after publication. Data associated with any ongoing development program will be made available within 6 months after approval of the relevant product. Requests must include a research proposal clarifying how the data will be used, including proposed analysis methodology. Research proposals will be evaluated relative to publicly available criteria available at https://www.biomarin.com/publication-data-request/ to determine whether access will be given, contingent upon execution of a data access agreement with BioMarin Pharmaceutical Inc.

## Abbreviations

AGV: annualized growth velocity
A/P: anterior/posterior
FGFR3: fibroblast growth factor receptor 3
IPD: interpedicular distance
L1-5: lumbar vertebra 1-5
LSM: least-squares mean
T1-5: thoracic vertebra 1-5 L1-5
TLK: thoracolumbar kyphosis
